# AI chatbots for promoting healthy habits: Legal, ethical, and societal considerations

**DOI:** 10.1177/20552076251390004

**Published:** 2025-11-10

**Authors:** Hannah van Kolfschooten, João Gonçalves, Nic Orchard, Caroline Figueroa

**Affiliations:** 1 Amsterdam Law School, 1234 University of Amsterdam, Amsterdam, Netherlands; 2 Center for Life Sciences Law, 27209 University of Basel, Basel, Switzerland; 3 Department of Media & Communication, 6984 Erasmus University Rotterdam, Rotterdam, Netherlands; 4 Faculty of Technology, Policy and Management, Delft University of Technology, Delft, The Netherlands

**Keywords:** Artificial intelligence, public health, chatbots, prevention, world health organization

## Abstract

Machine learning-based artificial intelligence (AI) chatbots are increasingly used to promote health and encourage individuals to adopt healthier behaviors. Chatbots driven by generative AI (genAI) simulate human interactions through text or voice to generate personalized content with guidance on topics such as smoking cessation, nutrition, managing stress, and sleep improvement. The use of AI chatbots for health promotion and wellness has been growing since 2023. While empirical evidence suggests their effectiveness in supporting behavioral change and mental health, the legal, ethical, and societal implications remains largely unexplored. This article presents a qualitative case study of S.A.R.A.H. (Smart AI Resource Assistant for Health), a genAI chatbot developed by the World Health Organization (WHO), analyzed against the six ethical principles outlined in the WHO's 2021 Guidance on Ethics and Governance of AI for Health. We also gathered exploratory insights from adolescent focus groups. These findings are descriptive and not based on formal thematic analysis. Drawing on this analysis, we identify key gaps between high-level ethical principles and practice and offer policy recommendations to guide responsible use of AI chatbots for health promotion.

## Introduction

Machine learning (ML)-enabled conversational agents, or artificial intelligence (AI) chatbots, are increasingly deployed by researchers, companies, and public health bodies to support behavioral changes, ranging from smoking cessation^1^ to improved diet,^2^ mental health,^3^ and sleep.^4^

Generative AI (genAI) chatbots powered by large language models (LLMs) simulate human conversation and enable more dynamic, context-sensitive interactions than earlier scripted systems.^5^ Since 2024, their popularity has surged alongside growing public adoption. While evidence of their effectiveness and cost-efficiency remains limited, early research suggests LLM-based chatbots may prevent non-communicable diseases, expand access to health information, especially in underserved populations, and reduce strain on health systems.^6^

Yet, the rapid deployment of these tools raises pressing questions about their ethical, legal, and societal implications. Are core principles such as autonomy, equity, and transparency upheld? How is user privacy protected? And can such systems truly deliver on their health-promoting promises? To explore these questions, we analyzed S.A.R.A.H. (Smart AI Resource Assistant for Health), an LLM-enabled chatbot launched by the World Health Organization (WHO) in 2024,^7^ using the WHO's own 2021 ethical AI principles outlined in the Guidance on Ethics and Governance of AI for Health.^8^ Focus groups with adolescents provided further insights into usability, trustworthiness, and value. We conclude with recommendations for ethical governance of genAI chatbots for promoting healthy behavior.

## The current state of AI chatbots for health promotion

Healthcare chatbots have long provided basic health information, answered common questions, and assisted with administrative tasks. These early systems relied on rule-based logic with scripted, inflexible responses. Recent advances in ML, particularly the rise of LLMs, have transformed chatbots into more dynamic, personalized, and interactive tools. Table 1 provides an overview of the evolution from early rule-based chatbots to current genAI systems used in health promotion.

**Table 1.** The evolution of health chatbots: traditional versus AI approaches.

	AI (ML) chatbots	Traditional chatbots
Features	Generates responses dynamically based on contextual understanding	Selects responses from a fixed set of pre-defined answers
Technology	Uses ML technologies like LLM and Computer Vision	Follows pre-defined rules and decision trees
Flexibility	Can learn from interactions and improve through re-training; Adapts to new situations and user preferences	Cannot learn or improve beyond pre-defined rules; Limited to scenarios covered by pre-defined rules
Interaction	Mimics human-like conversation with personalized responses	Scripted responses with minimal flexibility

The growth of genAI has accelerated the adoption of health chatbots. Notable examples include Ada Health and Buoy Health (symptom checkers) and Headspace (integrated CBT techniques). Specialized chatbots are also emerging, such as Daleela for Arabic-speaking women's health and Troomi, designed for adolescents.^9^

Early evidence suggests conversational AI can effectively support behavior change. A 2023 systematic review reported improvements in physical activity, diet, and sleep quality,^10^ while other studies noted that LLM-based chatbots (e.g. ChatGPT, Google Bard, LLaMA-2) can encourage healthy habits, though impact is greater among motivated users.^11^ Many users also value chatbots’ non-judgmental tone, empathy, and round-the-clock availability.^12^ Despite these benefits, AI chatbots pose risks to their users. We highlight these below, using the case study of S.A.R.A.H. as an example.

## Promises and pitfalls of genAI chatbots for public health

### Case study of S.A.R.A.H.: the WHO's digital health assistant

In March 2024, the WHO launched S.A.R.A.H., a genAI chatbot developed with Soul Machines (New Zealand).^13^ Designed as a research prototype, S.A.R.A.H. tests the feasibility of deploying genAI for health promotion at scale, offering 24/7 multilingual conversations on topics such as nutrition, stress management, and tobacco cessation. As the WHO's first experiment with genAI for public health communication, S.A.R.A.H. represents a valuable governance case study: it illustrates both the promise of AI-enabled health outreach and the complex ethical, legal, and societal challenges these technologies raise as they become integrated into public health strategies worldwide.

**Figure 1.**
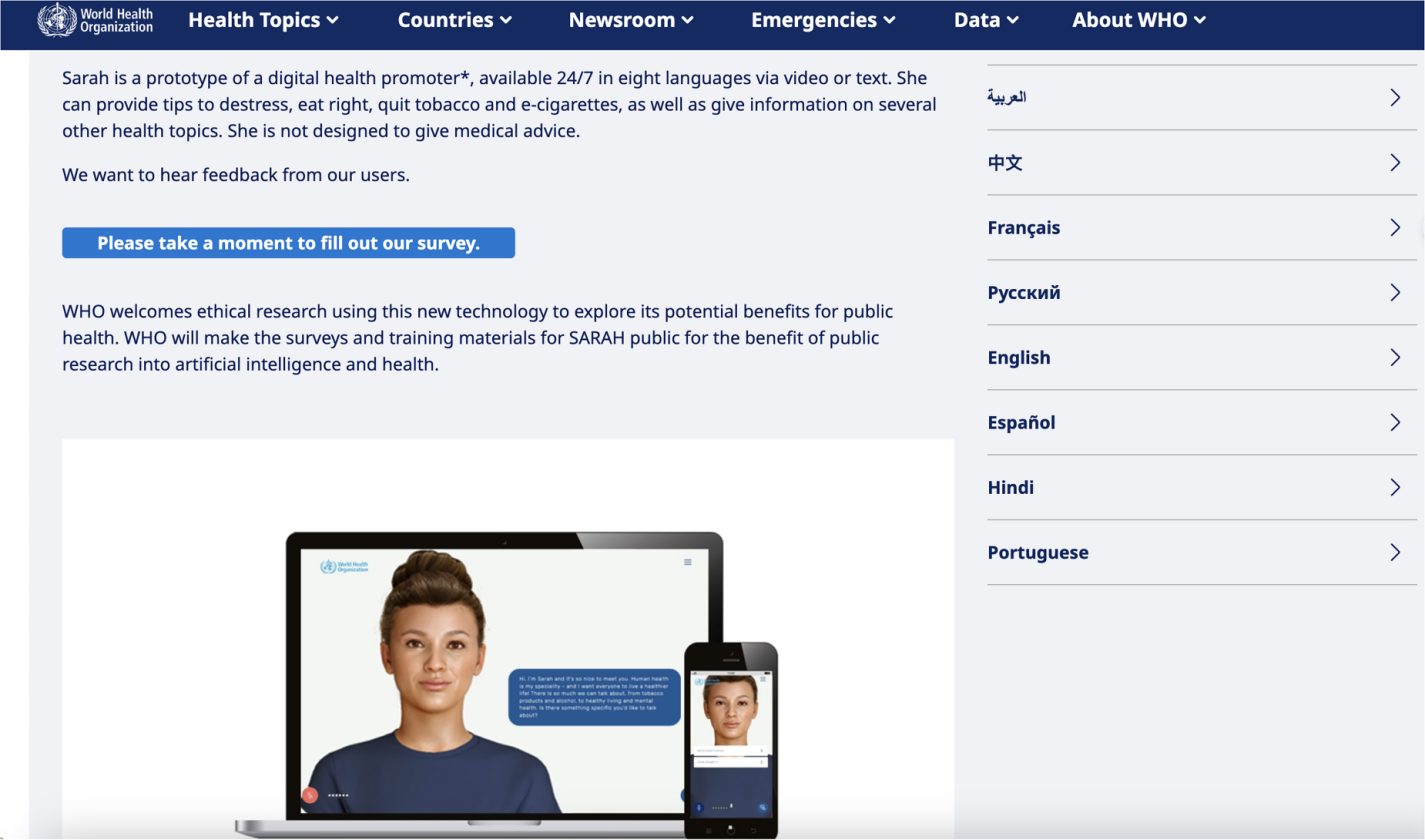
Screenshot of the WHO website describing chatbot S.A.R.A.H.

**Figure 2.**
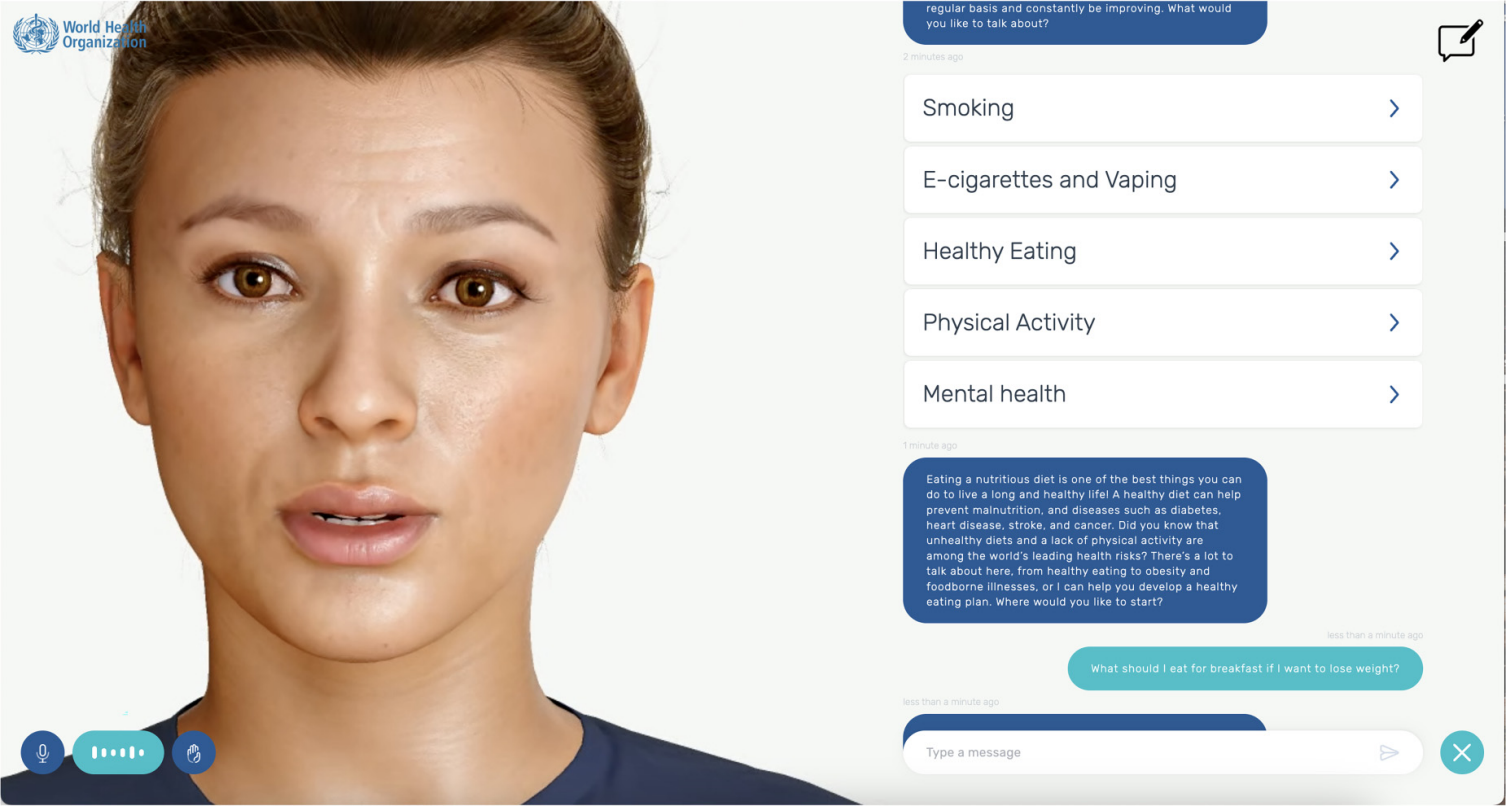
Screenshot of a sample conversation with S.A.R.A.H. about nutrition advice.

We analyze S.A.R.A.H. against the six ethical principles outlined in WHO's 2021 Guidance on Ethics and Governance of AI for Health^8^ and draw on user insights from adolescent focus groups. Figure 1 shows the general description of S.A.R.A.H. as provided by the WHO and lists the eight available use languages. Notably, it reveals how the WHO is explicitly asking for feedback and ethical research on their tool. Figure 2 is a screenshot of a sample conversation with S.A.R.A.H., in this case, about healthy eating habits. It shows how the tool uses facial expressions and records audio, but can also be used for written conversation. It lists the different areas of public health that the tool can assist users with.

### Methods

Our analysis combined three approaches. First, we interacted with S.A.R.A.H. to assess its usability and alignment with ethical principles. We documented its responses, privacy notices, and interface design. Second, we reviewed available documentation on its design, intended use, and safeguards from WHO, Soul Machines, and OpenAI between January and April 2025. Third, we drew on focus groups with 25 adolescents (aged 16–23) recruited through community centers in Rotterdam, the Netherlands. These focus groups took place in the first quarter of 2025 and were part of a larger, pre-registered qualitative study on adolescents’ experiences with mental health and health-related apps, for which a full thematic analysis is being conducted.^14^ Participants used S.A.R.A.H. and shared feedback on usability, trust, and accessibility.

For this article, we used only the subset of discussions that addressed S.A.R.A.H., which was introduced during the sessions as an example of a genAI chatbot for health promotion. Because this material is limited in scope, we present these findings descriptively to illustrate user perspectives and complement our legal and ethical analysis, rather than as a standalone qualitative dataset.

1. Protecting autonomy

S.A.R.A.H. aims to enhance users’ autonomy by offering accessible health information on topics such as diet, exercise, tobacco cessation, and stress management. This supports individuals in recognizing risk factors for major conditions like cancer, heart disease, and diabetes. However, autonomy is limited by the chatbot's tendency to offer generic advice unless explicitly prompted for tailored guidance. Adolescents in our focus groups noted that S.A.R.A.H.'s responses felt overly broad, sometimes resulting in advice that was either too general or poorly suited to their needs.

Privacy and consent issues further complicate autonomy. S.A.R.A.H. encourages users to enable camera and microphone access for a more “interactive experience,” capturing sensitive biometric data such as voice and facial expressions. While the WHO states that all conversation data is anonymized, multiple privacy policies apply (WHO, Soul Machines, OpenAI), creating a complex, fragmented framework. Users must navigate three separate privacy policies, none of which clearly describe how data flows between entities, where it is stored, or whether it is used for model training. This patchwork approach undermines informed consent and accountability, particularly given WHO's extraterritorial status and the cross-border nature of the platform's infrastructure.

**Figure 3.**
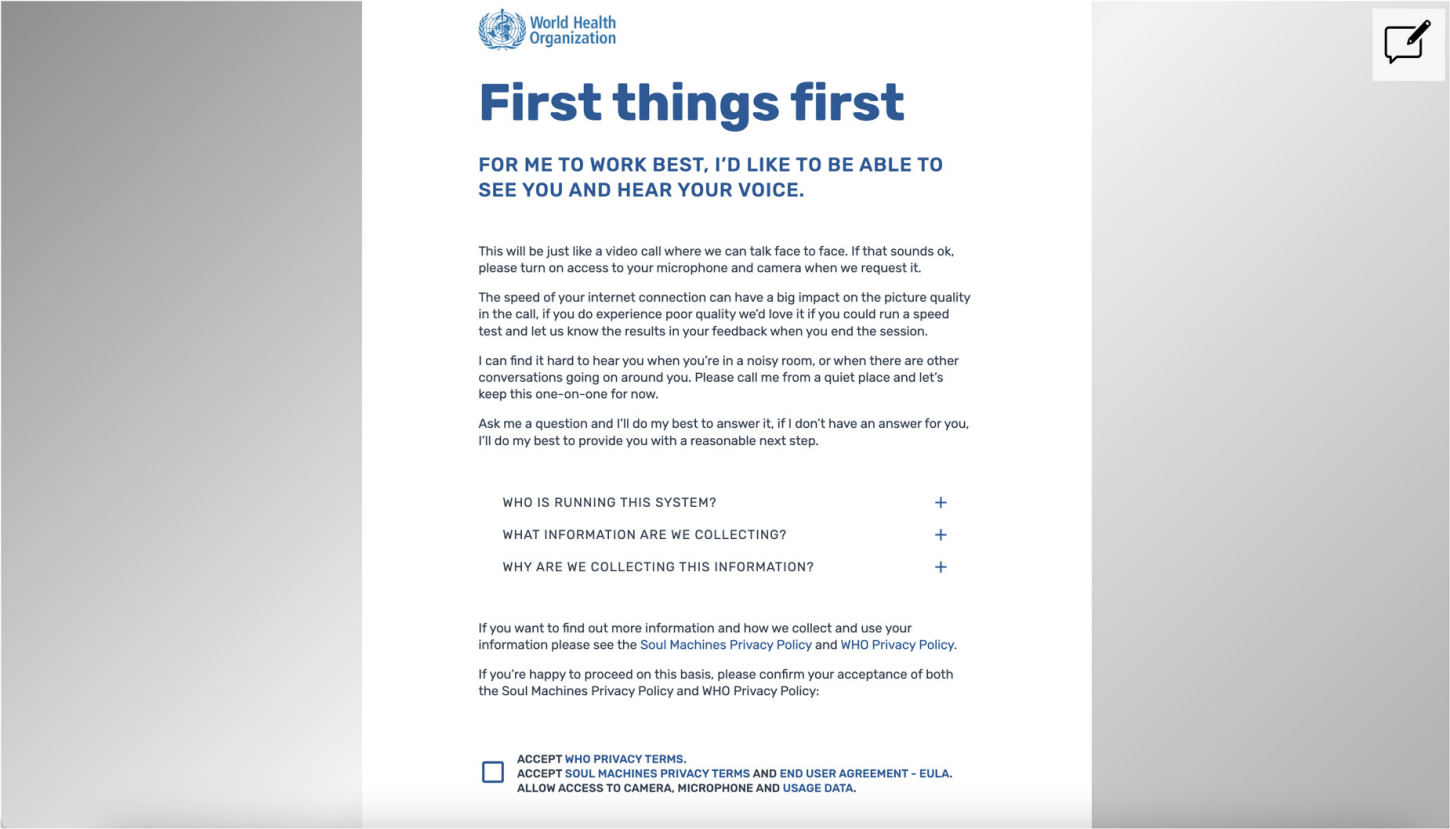
Screenshot of the information the user is given before accessing the S.A.R.A.H. chatbot.

Notably, S.A.R.A.H. provides no clear answers to users asking about how their data is stored, shared, or secured. In focus groups, adolescents expressed discomfort with the avatar “watching” and “listening,” which discouraged them from fully engaging with the tool. Without stronger privacy protections and clearer communication, AI chatbots like S.A.R.A.H. risk undermining user autonomy. Figure 3 shows the information the user is given before accessing the AI chatbot.

2. Promoting human well-being, human safety and the public interest

The WHO emphasizes that AI systems must avoid harm and ensure user safety, yet key safeguards appear to be missing in S.A.R.A.H. There are no age checks or warnings, leaving minors exposed to content not designed for them. The chatbot's authoritative tone and polished, human-like avatar risk fostering misplaced trust, with users potentially overestimating its expertise.^15^

Adolescents in our focus groups described the chatbot's answers as overly long, repetitive, and at times glitchy or incoherent, echoing broader research showing AI chatbots often struggle to maintain sensitive, fluid conversations. A critical gap is the lack of human oversight: when users disclose suicidality, S.A.R.A.H. advises seeking clinical help but offers no concrete resources. Generic advice without actionable support may heighten risks, especially where access to care is limited.^16^

While WHO guidance calls for continuous monitoring, it remains unclear how the organization ensures ongoing quality control, particularly given that development and maintenance have been outsourced. Without strong oversight and clear pathways for improvement, S.A.R.A.H.'s ability to uphold safety is uncertain.

3. Ensuring transparency, explainability, and intelligibility

The WHO emphasizes transparency in its ethical AI principles, yet S.A.R.A.H.'s implementation reveals important gaps. While the chatbot notes that training data can be made available on request, users are not informed about the specific language model powering its responses. Although the landing page links to OpenAI's privacy policy, it is not made explicit whether OpenAI's models are used, creating potential confusion.

S.A.R.A.H. assures users that conversation data are anonymized and points to privacy policies from WHO, Soul Machines, and OpenAI. However, users are not given the option to opt out of data collection for training purposes, nor are they informed in accessible language about how their data flow between these actors. Our focus group participants noted frustration when the chatbot declined to answer questions about its own privacy safeguards, undermining trust. Despite WHO's leadership in setting AI ethics standards, these shortcomings illustrate the difficulty of translating high-level principles into concrete, user-facing transparency.

4. Fostering accountability

The WHO endorses S.A.R.A.H. but disclaims responsibility for its content, warning users not to rely on its responses as medical advice or even as factually correct. This disclaimer, though legally sound, may undermine trust, especially given the high expectations users have of WHO-affiliated tools. Accountability structures are also minimal. Users can submit feedback via a general survey, but there is no clear process to report harmful content or engage with a responsible team. This lack of direct oversight weakens responsiveness to errors or risks.

Moreover, S.A.R.A.H. operates outside formal clinical care frameworks, meaning it is not subject to medical device regulations or legal protections tied to patient care, such as medical secrecy or liability standards.^17^ This regulatory gray area highlights an urgent need for clearer governance of AI health promotion tools, particularly when they carry the weight of institutional endorsement by the WHO.

5. Ensuring inclusivity and equity

Conversational AI holds promise for advancing health equity by offering 24/7 access, multilingual support, and adaptable formats. S.A.R.A.H. is available in eight languages, can be accessed via text or voice, and requires no app download, features that make it broadly accessible. However, our focus groups highlighted usability barriers for some groups, including elderly users and people with disabilities, who may struggle with its interface.

Personalization remains a challenge. S.A.R.A.H.'s initial responses often assume users are neurotypical and from high-income contexts; tailored advice requires explicit prompting. This risks alienating users with different needs or backgrounds. Bias is an inherent risk in AI health tools.^18^ While S.A.R.A.H. appears designed to offer culturally specific information, such as regional foods, details on training data and mitigation strategies are lacking. Without transparency on bias checks or safety audits, it is difficult to assess how effectively WHO is addressing equity concerns, underscoring the need for clearer safeguards in future implementations.

6. Responsive and sustainable AI

Responsive AI requires ongoing monitoring to ensure quality and safety in real-world use. For S.A.R.A.H., we found no evidence of active monitoring or mechanisms for users to flag issues, raising questions about how WHO oversees performance. Outsourcing development to private firms may further limit WHO's ability to audit or adapt the tool over time. Sustainability also means aligning AI with broader health system goals and minimizing environmental impact. It remains unclear how S.A.R.A.H. integrates with existing health infrastructures or whether it contributes to sustainable healthcare delivery. To date, no clear standards or strategies have been outlined to ensure AI health tools like S.A.R.A.H. support long-term public health priorities.

Taken together, our findings illustrate a clear AI governance gap. Chatbots like S.A.R.A.H. operate outside established regulatory categories and rely on a patchwork of privacy policies and contracts across multiple actors, each under different legal regimes. This fragmented approach blurs accountability, complicates consent, and weakens protection for sensitive health data. As genAI becomes embedded in public health strategies, building coherent, enforceable governance frameworks is essential to safeguard trust and equity.

## The way forward

AI chatbots for public health promotion are advancing rapidly, but governance frameworks have not kept pace. Our analysis of S.A.R.A.H. reveals critical gaps between ethical principles and real-world implementation, particularly concerning privacy, transparency, accountability, equity, and sustainability. Importantly, this case study is not just about one tool; it serves as a bellwether for the broader field of AI-driven health promotion. Why does this matter? First, S.A.R.A.H. is a flagship initiative by the WHO, a globally trusted authority in health. Its launch signals that genAI is moving from experimental to mainstream use in public health. Second, because the WHO was a key architect of global ethical AI standards, its own struggles to align S.A.R.A.H. with those principles expose fundamental governance and technical challenges that will confront many other actors worldwide. This case study, therefore, offers a critical, timely opportunity for researchers and policymakers to understand where existing frameworks fall short and how to improve them before widespread adoption.

The EU AI Act, which was adopted in 2024, offers one route to stronger oversight. It introduces specific obligations for “high-risk” AI systems, including health applications, and transparency requirements for general-purpose AI models, like the LLMs that underpin chatbots such as S.A.R.A.H. However, while promising, the AI Act alone will not be enough. It must be complemented by sector-specific rules that clarify when AI chatbots for health promotion fall within medical device regulation, as well as mechanisms for continuous audit and enforcement.^19^ The High-Level Expert Group on AI's “Ethics Guidelines for Trustworthy AI” already outlines key requirements, such as human agency, technical robustness, and societal well-being, that echo WHO's principles. Yet, as our analysis of S.A.R.A.H. shows, these principles remain aspirational without clear accountability, legal standards, and binding obligations. In this light, we recommend the following policy actions, summarized in Table 2 below:

1. **Establish clear legal classifications:** Governments and international health organizations should define when AI health chatbots qualify as medical devices and subject them to pre-market testing, certification, and post-market surveillance.
2. **Mandate transparency and disclosure:** Developers should be required to disclose the AI model's architecture, training data sources, and safeguards against bias and misuse. Privacy policies must be streamlined and written in plain language, with clear opt-outs for data use in further model training.^20^
3. **Strengthen data protection and privacy:** Chatbots must include age verification, prohibit commercial exploitation of health data, and provide clear, accessible consent mechanisms. Oversight bodies should audit compliance regularly.
4. **Enhance accountability and redress:** Clear liability frameworks must be established to clarify who is responsible when harm occurs. Mechanisms for user feedback, redress, and rapid response to harmful content should be standard features.
5. **Promote inclusivity and mitigate bias:** Developers must use diverse, representative datasets and ensure design choices (e.g. language options, accessibility features) accommodate varied user needs.^21^ Regular independent audits should be mandated to detect and correct bias.
6. **Integrate AI into broader health systems**: AI chatbots should be viewed as supplements to, not substitutes for, human-led care. Public health strategies should explicitly define the role of AI tools and ensure they are embedded within existing care pathways and community initiatives.^22^
7. **Support continuous oversight and sustainability:** AI chatbots should only be deployed after clear safeguards are established, including protocols for data protection, content accuracy, usability testing, and escalation pathways for users in crisis. Independent panels should be established to monitor AI chatbot performance in real time,^23^ and environmental standards for digital health tools should be developed to minimize carbon impact.

**Table 2.** Ethical challenges and policy responses for AI chatbots in health promotion.

Ethical principle	Key concerns with AI chatbots	Recommended actions
Autonomy	Fragmented privacy policies; unclear consent; weak protection for vulnerable users; generic advice limits informed choices.	Strengthen privacy policies and consent; implement age verification and safeguards; prioritize high-quality, contextual information; ensure chatbots complement human-led care.
Human well-being and safety	No age checks; human-like design fosters over-trust; limited crisis response; glitches and poor responsiveness; lack of human oversight.	Mandate human oversight for high-risk topics; require clear crisis resources; establish safety checks and continuous performance monitoring.
Transparency and explainability	Opaque data practices; unclear underlying models; inaccessible or confusing privacy disclosures; chatbot avoids privacy questions.	Enforce transparency on model architecture, training data, and safeguards; require simple, user-friendly privacy notices and opt-out options for data reuse.
Accountability	Minimal user reporting channels; unclear legal liability.	Define legal liability frameworks; create direct user reporting and redress channels; require regular independent audits and clear accountability structures.
Inclusivity and equity	Usability barriers for elderly and disabled users; default assumptions of neurotypical, high-income users; unclear bias mitigation.	Use diverse, representative datasets; ensure accessible, culturally appropriate design; mandate regular bias audits and transparent reporting of mitigation strategies.
Responsive and sustainable AI	No evidence of ongoing monitoring; unclear integration with health systems; unknown environmental impact.	Require continuous monitoring and evaluation; clarify chatbot roles in health system strategies; develop standards for the environmental sustainability of digital health tools.

Promising initiatives are emerging. A recent example is a chatbot for maternal health developed in southern Africa, trained in Sesotho, Shona, Ndebele, and English, to expand access to reliable maternal health information in underserved, resource-constrained communities.^24^ In Australia, researchers co-designed a chatbot with people waiting for eating disorder treatment to deliver single-session therapy.^25^ These examples suggest that effective, ethical deployment is possible if governance keeps pace with innovation. Our analysis of S.A.R.A.H. illustrates the stakes. This case shows that even the world's leading health authority struggles to meet its ethical commitments when deploying cutting-edge AI. For researchers and policymakers alike, S.A.R.A.H. provides an instructive example of both the opportunities and the pitfalls of using genAI in public health. It underscores the need to move beyond broad principles and put enforceable, well-designed governance structures in place.

## Limitations

This article is a case study that uses the WHO's genAI chatbot S.A.R.A.H. as a lens to explore the legal, ethical, and societal implications of AI chatbots for promoting healthy habits. As such, it does not aim to evaluate the chatbot's clinical effectiveness or provide a comprehensive technical assessment of its design. The focus groups discussed here form part of a larger pre-registered study of adolescents’ experiences with mental health and health-related apps; this paper draws only on the subset of comments related to S.A.R.A.H. and presents them descriptively rather than through a full thematic analysis. This approach reflects our intention to use qualitative user feedback as illustrative context for a normative analysis rather than as standalone qualitative findings.

Because this work is centered on legal, ethical, and governance considerations, its conclusions are not intended to be generalizable beyond the case of S.A.R.A.H. or to offer definitive judgments on all health chatbots. Instead, the case study provides insight into broader challenges in the design, regulation, and implementation of AI-driven health promotion tools, particularly those endorsed by trusted institutions like the WHO. Future research should complement this perspective with formal qualitative analyses of user experiences, empirical studies on chatbot safety and equity, and comparative legal analyses of regulatory approaches across jurisdictions.

## Conclusions

AI chatbots for health promotion offer new opportunities to make health information more accessible and responsive. They provide scalable, around-the-clock advice in multiple languages and formats, which could benefit underserved populations. This case study of S.A.R.A.H. provides exploratory insights into both the promise and the risks of using these tools to promote healthy habits. While our findings are based on a single chatbot and a descriptive subset of focus group data, they highlight important legal, ethical, and societal considerations, including concerns about privacy, transparency, accountability, equity, and sustainability. As a high-profile prototype backed by a trusted global health authority, S.A.R.A.H. provides enduring lessons for how genAI chatbots should be governed, tested, and deployed in health promotion.

To ensure AI chatbots contribute meaningfully to public health, stronger governance, clear legal requirements, and robust oversight are essential. Ethical principles must be translated into enforceable rules, and AI tools must be carefully integrated into public health strategies that center human expertise and community needs. Ongoing monitoring, transparency, and accountability are critical to keeping these tools safe and effective. If developed responsibly, AI chatbots can help close health gaps and promote better outcomes for diverse populations. However, as S.A.R.A.H. makes clear, there is much work to be done to ensure these tools meet their promise rather than deepen health inequities. Now is the time to build solid legal and ethical foundations before these technologies become more deeply entrenched.

## References

[1] Whittaker R Dobson R Garner K . Chatbots for smoking cessation: scoping review. J Med Internet Res 2022; 24: e35556. 10.2196/35556 PMC9514452 36095295

[2] Yang Z Khatibi E Nagesh N , et al. Chatdiet: empowering personalized nutrition-oriented food recommender chatbots through an LLM-augmented framework. Smart Health 2024; 32: 100465.

[3] Aggarwal A Tam CC Wu D , et al. Artificial intelligence–based chatbots for promoting health behavioral changes: systematic review. J Med Internet Res 2023; 25: e40789. 10.2196/40789 PMC10007007 36826990

[4] Rick SR Goldberg AP Weibel N . Sleepbot: encouraging sleep hygiene using an intelligent chatbot. In: *Proceedings of the 24th International Conference on Intelligent User Interfaces (IUI ‘19 Companion)*, Los Angeles, CA, 17–20 March 2019, pp. 107–108. New York: ACM.

[5] Ning Y Teixayavong S Shang Y , et al. Generative artificial intelligence and ethical considerations in health care: a scoping review and ethics checklist. Lancet Digit Health 2024; 6: e848–e856. 10.1016/S2589-7500(24)00143-2 PMC11542614 39294061

[6] Singh B Olds T Brinsley J , et al. Systematic review and meta-analysis of the effectiveness of chatbots on lifestyle behaviours. NPJ Digit Med 2023; 6: 1–10. 37353578 10.1038/s41746-023-00856-1 PMC10290125

[7] World Health Organization . WHO unveils a digital health promoter harnessing generative AI for public health. Report, World Health Organization, Geneva, 2 April 2024.

[8] World Health Organization . Ethics and governance of artificial intelligence for health: WHO guidance. Report, World Health Organization, Geneva, 2021.

[9] Abd-Alrazaq AA Alajlani M Alalwan AA , et al. An overview of the features of chatbots in mental health: a scoping review. Int J Med Inform 2019; 132: 103978. 31622850 10.1016/j.ijmedinf.2019.103978

[10] Bak M Chin J . The potential and limitations of large language models in identification of the states of motivations for facilitating health behavior change. J Am Med Inform Assoc 2024; 31: 2047–2053. 38527272 10.1093/jamia/ocae057 PMC11339501

[11] Siddals S Torous J Coxon A . “It happened to be the perfect thing”: experiences of generative AI chatbots for mental health. NPJ Ment Health Res 2024; 3: 1–9. 39465310 10.1038/s44184-024-00097-4 PMC11514308

[12] Kunze KN Jang SJ Fullerton MA , et al. What’s all the chatter about?: current applications and ethical considerations of artificial intelligence language models. Bone Joint J 2023; 105-B: 587–589. 37257860 10.1302/0301-620X.105B6.BJJ-2023-0156

[13] van Kolfschooten H . Towards an EU charter of digital patients’ rights in the age of artificial intelligence. DISO 2025; 4: 6.

[14] DOI: 10.17605/OSF.IO/KHFXU.

[15] Gerke S Babic B Evgeniou T , et al. The need for a system view to regulate artificial intelligence/machine learning-based software as medical device. NPJ Digit Med 2020; 3: 53. 32285013 10.1038/s41746-020-0262-2 PMC7138819

[16] Bharel M Auerbach J Nguyen V , et al. Transforming public health practice with generative artificial intelligence. Health Aff 2024; 43: 776–782. 10.1377/hlthaff.2024.00050 38830160

[17] van Kolfschooten H . The AI cycle of health inequity and digital ageism: mitigating biases through the EU regulatory framework on medical devices. J Law Biosci 2023; 10: lsad031. 10.1093/jlb/lsad031 PMC10709664 38075950

[18] Zack T Lehman E Suzgun M , et al. Assessing the potential of GPT-4 to perpetuate racial and gender biases in health care: a model evaluation study. Lancet Digit Health 2024; 6: e12–e22. 10.1016/S2589-7500(23)00225-X 38123252

[19] van Kolfschooten H van Oirschot J . The EU Artificial Intelligence Act (2024): implications for healthcare. Health Policy 2024; 149: 105152. 39244818 10.1016/j.healthpol.2024.105152

[20] Malgaroli M Schultebraucks K Myrick KJ , et al. Large language models for the mental health community: framework for translating code to care. Lancet Digit Health 2025; 7: e282–e285. 10.1016/S2589-7500(24)00255-3 PMC11949714 39779452

[21] Meyrowitsch DW Jensen AK Sørensen JB , et al. AI Chatbots and (mis)information in public health: impact on vulnerable communities. Front Public Health 2023; 11: 1226776. 38026315 10.3389/fpubh.2023.1226776 PMC10644115

[22] Taylor L . There Is an App for That: Technological Solutionism as COVID-19 Policy in the Global North. In: Aarts E, Fleuren H, Sitskoorn M, et al. (eds) New Common. Cham: Springer, 2021, pp.209–221.

[23] Aloise I Migliara G Franceschetti A , et al. Enhancing public health: training an AI chatbot for complex tasks support. Eur J Public Health 2024; 34: ckae144.1195.

[24] Batani J Mbunge E Leokana L . A deep learning-based chatbot to enhance maternal health education. In: *2024 Conference on Information Communications Technology and Society (ICTAS)*, 2024, pp. 7–11.

[25] Sharp G Dwyer B Xie J , et al. Co-design of a single session intervention chatbot for people on waitlists for eating disorder treatment: a qualitative interview and workshop study. J Eat Disord 2025; 13: 46. 40069853 10.1186/s40337-025-01225-x PMC11899673

